# Hybrid Brain-Computer-Interfacing for Human-Compliant Robots: Inferring Continuous Subjective Ratings With Deep Regression

**DOI:** 10.3389/fnbot.2019.00076

**Published:** 2019-10-10

**Authors:** Lukas D. J. Fiederer, Martin Völker, Robin T. Schirrmeister, Wolfram Burgard, Joschka Boedecker, Tonio Ball

**Affiliations:** ^1^Neuromedical AI Lab, Department of Neurosurgery, Medical Center – University of Freiburg, Faculty of Medicine, University of Freiburg, Freiburg, Germany; ^2^BrainLinks BrainTools, Cluster of Excellence, University of Freiburg, Freiburg, Germany; ^3^Graduate School of Robotics, University of Freiburg, Freiburg, Germany; ^4^Department of Computer Science, University of Freiburg, Freiburg, Germany

**Keywords:** robot behavior, autonomous robots, BCI, regression, CNN, deep learning, random forests, support vector machines

## Abstract

Appropriate robot behavior during human-robot interaction is a key part in the development of human-compliant assistive robotic systems. This study poses the question of how to continuously evaluate the quality of robotic behavior in a hybrid brain-computer interfacing (BCI) task, combining brain and non-brain signals, and how to use the collected information to adapt the robot's behavior accordingly. To this aim, we developed a rating system compatible with EEG recordings, requiring the users to execute only small movements with their thumb on a wireless controller to rate the robot's behavior on a continuous scale. The ratings were recorded together with dry EEG, respiration, ECG, and robotic joint angles in ROS. Pilot experiments were conducted with three users that had different levels of previous experience with robots. The results demonstrate the feasibility to obtain continuous rating data that give insight into the subjective user perception during direct human-robot interaction. The rating data suggests differences in subjective perception for users with no, moderate, or substantial previous robot experience. Furthermore, a variety of regression techniques, including deep CNNs, allowed us to predict the subjective ratings. Performance was better when using the position of the robotic hand than when using EEG, ECG, or respiration. A consistent advantage of features expected to be related to a motor bias could not be found. Across-user predictions showed that the models most likely learned a combination of general and individual features across-users. A transfer of pre-trained regressor to a new user was especially accurate in users with more experience. For future research, studies with more participants will be needed to evaluate the methodology for its use in practice. Data and code to reproduce this study are available at https://github.com/TNTLFreiburg/NiceBot.

## 1. Introduction

### 1.1. Brain and Non-brain Signals

In brain-computer interfaces (BCIs) for the control of assistive robots, a safe and human-compliant behavior of the robot during the interaction with its user is a crucial factor. However, what behavior is assessed as “safe” depends strongly on subjective parameters (Feil-Seifer et al., 2007). For example, users might differently react to robot movement at higher or lower speeds, or robotic poses in proximity to the user's body or face. Moreover, users might perceive the robot's behavior in different ways depending on personal variables, e.g., their previous exposure to robots. In this study, we describe a BCI-compatible method to continuously acquire subjective rating data about the quality of robotic behavior in real-time during a human-robot interaction task. The real-time nature and BCI compatibility are crucial as, combined with the measurement of electroencephalography (EEG), electrocardiography (ECG) and respiration, we aim to identify inter-personal commonalities and differences, specific rating strategies, and their stability over time. Further, we evaluate regression techniques to allow an automatic prediction of subjective ratings, which could be used to automatically adapt the robot's behavior to user-specific preferences using reinforcement-learning. We extend the traditional hybrid BCI framework (Pfurtscheller et al., 2010), combining brain and non-brain signal, by including information from the robot into the regressions.

### 1.2. Related Work

In the field of human-robot interaction, the assessment of robotic behavior has been a key part in a number of studies. Huang and Mutlu (2012) developed a toolbox for behavioral assessment of humanoid robots. There, the authors focus especially on human-like social behavior in robots. Ratings for variables of robot behavior, e.g., naturalness, likability, and competence, were collected after the experiments rather than during the actual interaction. Tapus et al. (2008) proposed a robot personality matching for robot behavior adaptation in post-stroke rehabilitation. To adapt the robots behavior, the authors used a Policy Gradient Reinforcement Learning (PGRL) Algorithm. The robot collected feedback from the user with voice recognition, using discrete classes such as “yes,” “no,” and “stop.” Sekmen and Challa (2013) combined sensory input from speech recognition, natural language processing, face detection and recognition, and implemented a Bayesian learning mechanism to estimate and update a parameter set that models behaviors and preferences of users. Specifically, they predict future actions of their users to prepare the robot for these. In a recent study of Sarkar et al. (2017), the effects of robot experience and personality of a user on the assessment of, among other factors, trust into the robot were assessed. Interestingly, the group of participants with previous robot experience rated their safety during the interaction with the robot on a lower level than the group which had no previous experience with robots. Less experienced people also rated the robot as more intelligent in this study.

Relevant to the decoding of perceived danger from EEG data, Kolkhorst et al. (2017) decoded the perceived hazardousness in traffic scenes from EEG data. This could also be used in human-robot interactions to prevent potentially dangerous situations. Kolkhorst et al. (2018) further developed an EEG-based target selection in collaboration with robotic effectors, which could harmonize well with assessment of robot behavior in human-machine interactions. Ehrlich and Cheng (2018) recently developed a system to validate robot actions by decoding error-related signals from EEG. Related to this, a number of studies in recent years have shown that the performance of robots in BCI scenarios can be enhanced with error decoding, e.g., in shared-control BCIs (Iturrate et al., 2013), or during the observation of autonomous robots (Salazar-Gomez et al., 2017).

In recent years, promising new approaches to decoding information from brain signals for BCI control were developed, e.g., deep learning with convolutional neural networks (CNNs). A major advantage of CNNs is that feature extraction and classification are combined into a single learning process, removing the need to manually extract features. After pioneering achievements in the field of computer vision, they are increasingly being adapted to problems of EEG decoding (Manor and Geva, 2015; Bashivan et al., 2016) and are the subject of active research (e.g., Eitel et al., 2015; Watter et al., 2015; Oliveira et al., 2016). These biologically inspired networks have a great potential to improve the accuracy of BCI applications (Burget et al., 2017; Schirrmeister et al., 2017; Kuhner et al., 2019). They additionally can be applied to the raw EEG data, greatly simplifying the design of BCI pipelines. We further demonstrated the usefulness of CNNs for error decoding from noninvasive (Völker et al., 2018c) and intracranial EEG (Völker et al., 2018b).

In contrast to discrete decoding problems, regression analysis with neural networks have become more popular in the recent time. Most use cases shown so far applied regression methods to video or image data. For example, Held et al. (2016) used regression to successfully track objects in videos at 100 frames per second. Shi et al. (2016) presented a regression approach to identify facial landmarks to subsequently align faces in images. In order to detect and localize robotic tools during robot-assisted surgery, Sarikaya et al. (2017) implemented a regression layer into a CNN. Miao et al. (2016) used regression techniques for a real-time 2D and 3D registration of X-ray images. With a CNN regressor, Viereck et al. (2017) improved the accuracy of robotic grasping and object recognition with respect to simulated depth images.

### 1.3. Aims and Objectives

**Figure 1:**
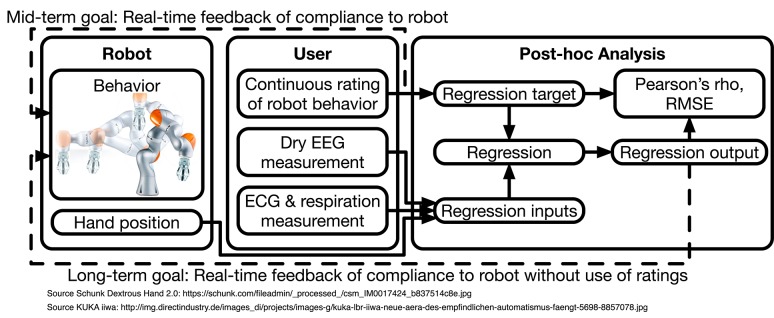
Structural diagram. A robot performs pre-programmed trajectories in the proximity of a user. The user subjectively and continuously rates the human compliance of the trajectories. In parallel, EEG, ECG, and respiration are measured. After the experiments, the EEG, ECG, respiration, and hand position data are used to regress the ratings. We report the correlation coefficient (Pearson's ρ) and root mean square error (RMSE) between ratings and regression outputs as intuitive quantitative measures. Our long-term goal is to provide real-time feedback to reinforcement-learning algorithms controlling the robot without explicit user ratings.

The goal of this study was to assess the feasibility of acquiring continuous data on the subjective perception of the behavior of assistive robots in a BCI context. Considering this context, the approach should not be limited to robots of a certain design, e.g., humanoid or not. Rather, we aimed to create a generalizable method for the subjective assessment of robot behavior, utilizable in real-time during BCI (and other) experiments. In future applications, such real-time ratings could then be leveraged for reinforcement-learning algorithms designed to adapt robotic behavior during the interaction in a human-compliant manner. Importantly, we wanted to evaluate subjective perception not by discrete values, but with a continuous rating system, allowing a more fine-grained analysis of the outcome. By recording EEG, ECG, and respiratory data simultaneously, we aimed to allow a search for physiological correlates of these ratings, which could later be used as input for an implicit situation assessment, without the user needing to explicitly rate the robot's behavior. Finally, we aimed to use regression methods to create an automatic and continuous prediction of subjective ratings for each user, and evaluate which kinds of input data and EEG features are the most informative about subjective perception during direct interaction with a robotic assistant. Our procedure is schematically summarized in Figure 1.

## 2. Experiments

**Figure 2:**
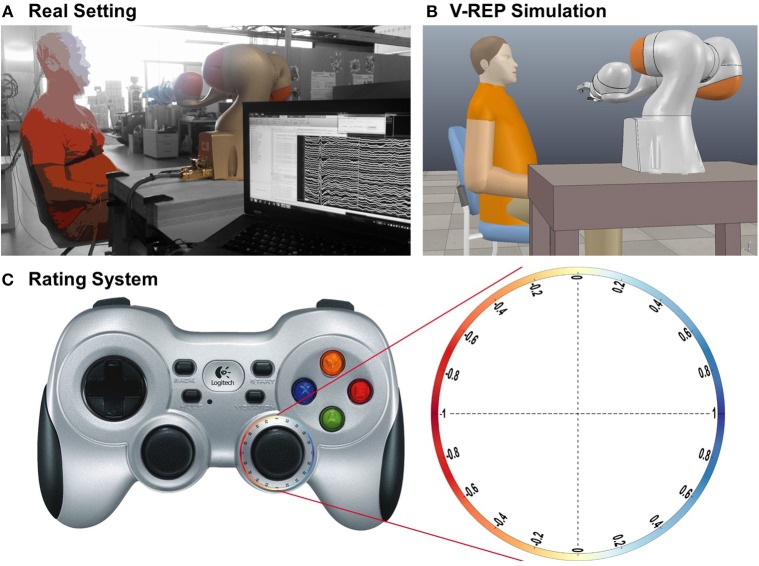
Paradigm setting, reconstruction, and rating system. **(A)** Photograph of the real setting. The picture was edited to make the user anonymous and to display the background in black and white. **(B)** Reconstruction in V-REP. **(C)** Robot behavior rating system with a wireless controller. The users rated the robot's behavior within a continuous range from −1 to 1. To do that, the users had to keep a thumbstick on a wireless controller on an outward circular path with their right thumb, where a left position represented a very bad rating (−1, red), a position to the right represented a good rating (1, blue), and a position straight up or down represented a neutral rating (0, yellow). This system was introduced so that the movement effort for the thumb was the same at each position, making an EEG analysis possible.

We conducted a series of experiments to evaluate robot behavior during interaction with an assistive robot grasping an object and delivering it to the user. The users were instructed not to move any body part during the experiment, to keep the interaction similar to that of a paralyzed person with a robot and to prevent muscle activity from contaminating the EEG data. Users were informed that the robotic arm would simulate the grasping of an object, e.g., a cup of water, and bring it forward toward them. The users did not have any prior knowledge about the path or velocity the robot would use. In some of the trajectories, the robot would deviate from the correct trajectory, e.g., by stopping in a wrong position, i.e., not in range of the user, or by positioning its hand above and behind the user's head. The trajectories were pre-programmed to ensure comparable experiments across all users. Figures 2A,B show the real and simulated experimental setup, respectively.

### 2.1. Hardware

As robotic arm, the LBR iiwa 7 R800 (KUKA Robotics), a 7 DOF lightweight robot combined with a three-fingered hand (Dexterous Hand 2.0, Schunk) was used. EEG was recorded using the g.SAHARA dry active electrode system and three g.USBamp amplifiers (Guger Technologies). Usable without electrode gel, dry electrodes have the advantage to be set up faster than systems with wet electrodes and may thus be more convenient for the user. The system used in this study is designed to capture a frequency range from 0.1 to 40 Hz. We recorded with 32 dry electrodes on the scalp positioned according to the 10-20 system at Fp1, Fpz, Fp2, AF7, AF3, AFz, AF4, AF8, F5, F3, F1, Fz, F2, F4, F6, FC1, FCz, FC2, C3, C1, Cz, C2, C4, CP3, CP1, CPz, CP2, CP4, P1, Pz, P2, and POz. The reference electrode was placed on the left mastoid, the ground electrode on the right mastoid. Further, ECG was recorded with two electrodes on the users' right clavicle and lowest left rib, and respiration was monitored with a respiration belt.

### 2.2. Users

Feasibility of continuous real-time rating of the subjective perception of robot behavior was evaluated in three users [age: 24 (S1), 26 (S2), and 30 (S3), all right-handed, S1 female]. S1 had no previous robot experience, S2 moderate experience with robots, i.e., worked with robots in a BCI context irregularly for approximately 2 years, and S3 already had a substantial amount of experience working with robots, i.e., worked extensively with robots at university and in in-depth projects for multiple years. All users were students of the University of Freiburg. Informed consent was provided before participation. The experiments were approved by the ethics committee of the University of Freiburg.

The users were seated in a way so that they could observe the robot's movements without moving their head, and in a position in which the trajectories of the robotic arm could not intersect in any way with the users' body or head. 45, 95, and 95 trajectories were recorded in block of 15 for each user, respectively.

### 2.3. Rating System

The users were instructed to rate the quality of the behavior of the robot continuously during their interaction by moving the right thumbstick on a wireless controller (Logitech F710) into different directions (Figure 2C). We did not ask for a more specific evaluation variable to gain a preferably generalizable evaluation of the robot's performance.

To rate the robot's behavior as good, the users had to move the thumbstick to the right; a position on the left was linked to a bad rating, and a position in the middle corresponded to a neutral rating. This rating strategy was thus designed in a way that, at all times, the users had to keep the thumbstick at a maximal deflection, to generate a tonic motor output at a similar level irrespective of the rating conveyed and thus to minimize movement-related brain responses possibly confounding EEG correlates of the ratings (see section 5). Randomizing the direction of rating for each robot trajectory could be used to further avoid such a confound but would make the task more difficult for the users and could thus potentially introduce inadvertently wrong ratings. We therefore chose to keep the direction of rating constant across all robot trajectories in this pilot study.

To calculate the rating, we first had to translate the x and y position of the thumbstick (both ranged from −1 to 1) into a rotation angle, and from that to a continuous rating from −1 (very bad) to 1 (very good), as shown in Figure 2. Thus, the conversion of the thumbstick x, y position to the rating is defined as

(1) $$\begin{array}{r} rating=\frac{abs\left(arctan2d\left(y,x\right)\right)-90}{90} \end{array}$$

where arctan2d is the four-quadrant inverse tangent in degrees.

### 2.4. Real-Time Data Processing

Robot joint angles were controlled with the MoveIt! motion planning framework (Chitta et al., 2012) via the Robot Operating System (ROS) (Quigley et al., 2009). EEG and peripheral (ECG, respiration) data were recorded at a 512-Hz sampling rate with the BCI2000 software (Schalk et al., 2004), using the Matlab Signal Processing (Matlab 2014a, The MathWorks, USA) module for real-time access to the raw EEG signals. In Matlab, a network connection to the local ROS master, controlling the robotic arm, was established. During the recording, the data were stored in a ring buffer and processed 16 times per second. EEG data were re-referenced to common average and filtered with a Butterworth band-pass filter of 3rd order between 0.5 and 40 Hz. For this, the filter coefficients were passed on between the blocks to avert filter artifacts and allow filtering on such short time segments. A ROS custom message type was used for the broadcasting of the data. The collected EEG, ECG, respiration, and the rating data were sent 16 times per second to the ROS master, where they were timestamped and stored together with the seven joint angles of the robotic arm.

### 2.5. *Post-hoc* Data Reconstruction

**Figure 3:**
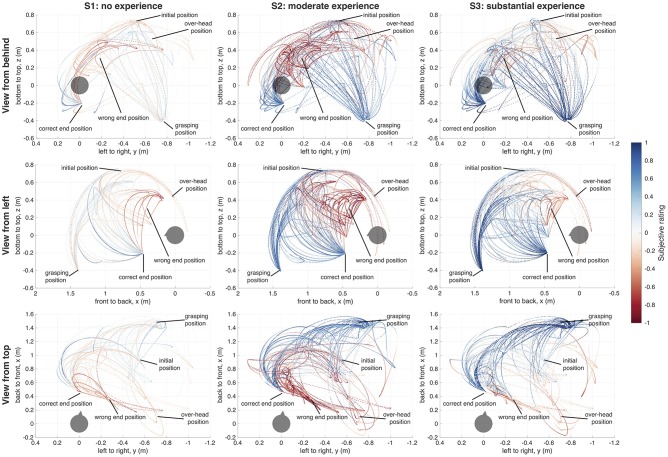
Effector trajectories and ratings. Users with no previous robot experience (S1), moderate robot experience (S2), and substantial robot experience (S3). The positions of the points correspond to the position of the base of the robotic hand. The color of each point displays the user's rating of the robot's behavior at this point in time. Red color indicates a negative rating, blue color indicated a positive rating; white corresponds with a neutral rating. The axes show the distance of the robotic hand from the user's head (gray sphere, triangle represents user's nose) in meters within the respective dimension (x, y, z).

Robot joint states, EEG, physiological recordings, and rating data were all stored in ROS bag files and later loaded into Matlab with help of ROS. To be able to reconstruct the exact trajectories of the robot, e.g., to calculate the distance between the users' head and the robotic hand and the hand's velocity, V-REP (Rohmer et al., 2013) (Coppelia Robotics) was used together with its Matlab API (Figure 2B). As replacement for the Schunk hand, which was not available in V-REP, we used the BarrettHand (BARRETT TECH) in the reconstruction, which has approximately the same size and shape, and was also equipped with three fingers. The trajectories, relative to the users head, are show in Figure 3.

### 2.6. Evaluation Structure

The data acquired during the pilot experiments is evaluated two-fold. First, to better understand the recorded data and investigate the influence of user robot experience, we analyse the data and present the results of this feature-driven analysis in section 3. To permit a richer analysis, the experiments were further recreated in reconstructions using V-REP, as described above. Second, to evaluate which kind of input data are potentially the most informative for proving feedback to reinforcement-learning algorithms, we perform end-to-end regression analyses of the data in the attempt to reconstruct the users' ratings of the robotic behavior. Regressions are performed within and across users. We quantify the regression results using correlations (Pearson's ρ) and the root mean square error (RMSE). These metrics are reported in section 4.5. Figure 1 schematically depicts the structure of our approach.

## 3. Trajectory and Rating

From the reconstruction in V-REP, we extracted the position of the robotic hand base over time. By combining the positions in 3D with the subjective rating at the same points in time, we were able to create trajectory-rating maps for each user, which are shown in Figure 3.

The trajectory-rating maps reveal a number of commonalities in the robot ratings across the users. For example, trajectories which were pre-programmed to end in an incorrect position (i.e., not within reach of the user) were expected and consistently labeled as “bad” by all users. Further, positions in which the robotic hand was above and behind a user's head, and thus not in the user's field of vision were also always rated as “bad.” The initial robot pose was mostly rated as neutral or close-to neutral. Other distinct positions of the robotic hand, like grasping, over-head, or correct/incorrect end-positions were rated more strongly, either positive or negative, than positions occurring during ongoing movements, or between the distinct poses.

However, despite the small number of users investigated, we also observed some distinct inter-individual differences in the robot ratings. S1, with no previous robot experience, gave in general lower ratings (mean: 0.04 ± 0.34), even if the robot was fulfilling the task objectively correct, like in the grasping position. The correct end-position was also sometimes rated as bad, especially if the robotic hand approached the user in a relatively steep angle from above. The negative ratings were often only in the range of 0 to −0.5. S2, with moderate robot experience, rated the majority of poses as good (mean: 0.23 ± 0.59), except the positions in which the robotic hand was positioned over the user's head, and thus out of the field of vision, and the wrong end-position. These were rated as strongly negative. S3, with a substantial amount of robot experience, again rated the robot's behavior as overall more positive than the other two users (mean: 0.37 ± 0.44). In general, the trajectory ratings in S3 appeared to be more similar to the user with moderate robot experience. Over-head and wrong end-position were rated negatively by all users, but least so by S3.

### 3.1. Key Poses During the Human-Robot Interaction

**Figure 4:**
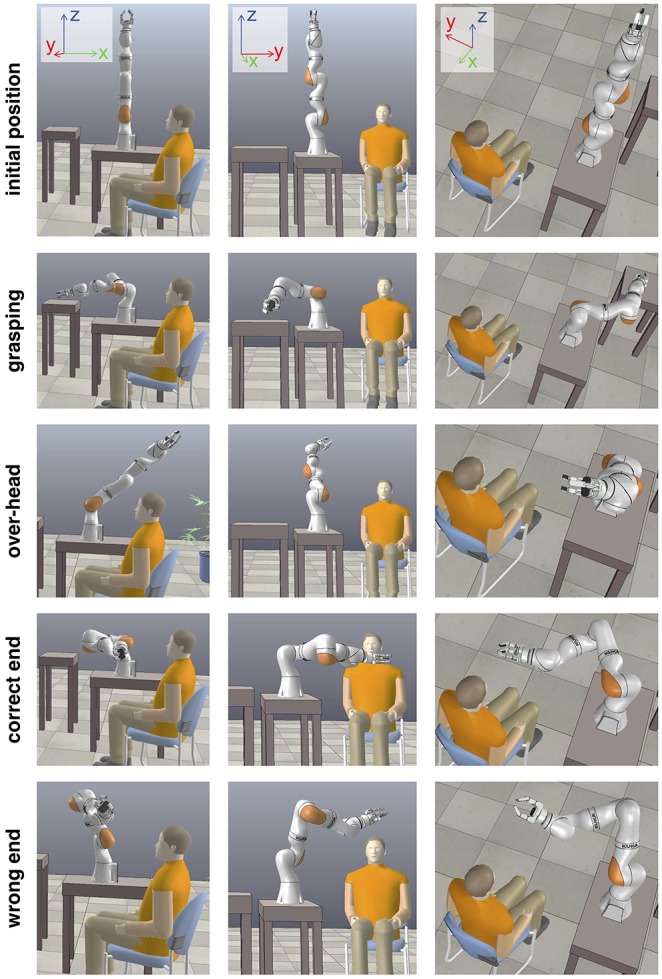
Key robot poses during the human-robot interaction. In addition to the initial pose, four key poses were pre-programmed into the trajectories. The object-grasping pose, a pose above the user's head and outside of its field of view, the correct end pose where the grasped object is delivered to the user, and the wrong end pose where the grasped object cannot be properly delivered to the user. Screenshots from the V-REP reconstruction.

From these trajectory-rating maps, we selected four key positions of the robot hand for deeper analysis: the grasping position, the over-head position, the correct end-position, and the incorrect end-position. These, and the initial position, are displayed from three different viewpoints in Figure 4.

**Figure 5:**
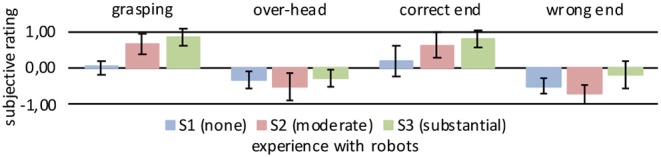
Mean subjective ratings at key robot positions. Key robot positions are depicted in Figure 4. Rating sample from cube with 40 cm side length centered on key pose. The error bars show the standard deviation of the rating sample.

In the four key positions, we calculated the mean rating for each user by identifying the spatial center of the robot hand position associated with each key pose and extracting the ratings within a cube with 40 cm side length around the center point. The mean ratings for the different users are listed in Table 1 and illustrated in Figure 5.

**Table 1:** Mean ± std subjective ratings at key robot positions.

**User (rob. exp.)**	**Grasping**	**Over-head**	**Correct end**	**Wrong end**
S1 (no)	0.00 ± 0.21	−0.33 ± 0.23	0.20 ± 0.42	−0.50 ± 0.21
S2 (moderate)	0.64 ± 0.27	−0.52 ± 0.38	0.63 ± 0.33	−0.70 ± 0.25
S3 (substantial)	0.84 ± 0.22	−0.28 ± 0.22	0.79 ± 0.25	−0.17 ± 0.38

### 3.2. Development of Ratings Over the Time of the Experiment

**Figure 6:**
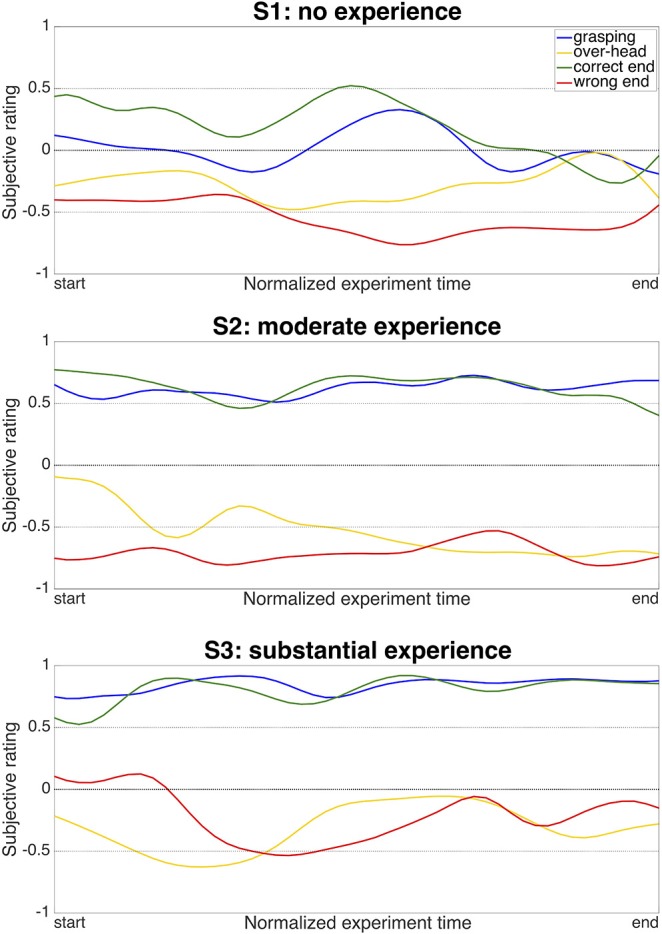
Development of ratings at key robot positions during the experiment. The y-axis shows the subjective rating when the robotic hand was positioned for grasping (blue), in an over-head position (yellow), at the correct end-position for delivery (green), or at a wrong, too-high end-position (red). The x-axis shows the development of the ratings over the duration of the experiment. The ratings are smoothed with a moving-average filter consisting of a Gaussian weighted window with the size of $\frac{1}{5}$th of the total samples per condition, which was convolved with the raw ratings.

As we expected that robot ratings might undergo systematic changes on the time scale of our experiment, we further analyzed the temporal development of ratings in these key positions (Figure 6). The long-term stability was assessed by applying a moving-average filter to smooth out fast fluctuations. While the overall distribution of ratings for the key positions stayed quite stable over the experiment, the absolute strength of the ratings did vary over the course of the experiment, especially in S1, the user without robot experience. Specifically, S1 displayed a tendency to rate the objectively “positive” poses, i.e., the grasping pose and the correct end-position, lower toward the end than at the beginning of the experiment. In S2 and S3, the users with moderate and substantial robot experience, particularly the ratings of the positive poses stayed more stable over time. Together, these observations fit well to our expectations that robot perception may change over time, and that such changes may be depended on previous robot exposure.

### 3.3. Effect of Robot Distance and Velocity

To understand the relationship of the subjective rating with both the velocity and the distance of the robotic hand in reference to the user's head, we calculated these metrics for each time point. For each user and feature, we estimated Pearson's Linear Correlation Coefficient (Pearson's ρ) (Table 2).

**Table 2:** Linear correlation of robot distance and velocity with subjective ratings.

	**Linear correlation with subjective rating**	
	**Robot hand distance**	**Robot hand velocity**
**User (rob. exp.)**	**Pearson's** **ρ**	
S1 (no)	−0.002	0.226
S2 (moderate)	0.369	−0.039
S3 (substantial)	0.205	−0.197

The distance of the robotic hand correlated moderately positive with the rating of the users S2 and S3, while it did not exhibit any correlation for S1 (with no previous robot experience). The correlation of subjective rating with the hand velocity was only weakly negative for S3, and moderately positive in S1, while there was no such effects in S2.

## 4. Regression for Rating Prediction

To show the feasibility of our proposed method we trained diverse regressors to predict the subjective ratings from the recorded data within and across users. We focused on the aforementioned feasibility, exploring features proposed in the literature and probing for a possible motor bias. Regressions are performed with python using pytorch (Paszke et al., 2017) version 1.0.0, braindecode (Schirrmeister et al., 2017) version 0.4.7 and scikit-learn (Pedregosa et al., 2011) version 0.20.2.

### 4.1. Data Pre-processing

For each user, we downsampled the data to 256 Hz, standardized to a mean of 0 and a variance of 1 using an exponentially weighted mean with factor 0.001 and split them three-fold. We kept the last three minutes of data separate for final evaluation of our regressors. The last 3 min before the final evaluation set were used as validation set during the manual hyper-parameter search. The remainder of the recording was used as training set. For the final evaluation, which is reported here, training set and validation set were combined into a single training set.

### 4.2. Evaluation Metrics

We evaluated the similarity of the predicted rating to the true rating by calculating their correlation coefficient (Pearson's ρ) and the RMSE of their difference. We report both metrics here because they reflect two different aspects of the predictions. The correlation coefficient only compares the shape of predictions and ratings while the RMSE compares the values. For reinforcement-learning, proper approximation of the shape of the rating is already sufficient. Using predicted ratings which additionally have correct scaling and value range is of course better but much more difficult to achieve. Thus, our preferred metric is the correlation coefficient.

### 4.3. Feature Extraction

To probe for differential information content, we split the data into different components. First the different modalities, the robot hand 3D coordinates (robot pos.), the ECG and respiration data (periphery data), and the EEG data were split. Each of these data modalities and their combinations were fed as-is to the regressors. To prevent to much redundancy in the results we only report the combination of all three modalities. For modality splits containing EEG data, we additionally tested three electrode selections and seven frequency-band selections. Based on literature (Cavanagh et al., 2010; Cavanagh and Frank, 2014; Spüler and Niethammer, 2015; Völker et al., 2018a), a potential target for a physiological brain signal underlying the rating could potentially be the delta (0–4 Hz) and theta (4–8 Hz) frequency bands, especially in the midline electrodes. Thus we, in addition to the raw EEG data, used band-passes from 0 to 4 and 4 to 8 Hz and selected all electrodes located on the head midline (all electrodes containing a z in the 10–20 nomenclature). To investigate the influence of a potential motor bias we further band-passed the EEG data from 8 to 14 Hz (alpha band), 14 to 20 Hz (low-beta band), 20 to 30 Hz (mid-beta band) and 30 to 40 Hz (high-beta/low-gamma band) and selected all electrodes located on the sensorimotor cortex (all electrodes containing a C in the 10–20 nomenclature). We report the results of all the triplet combinations of these features, i.e., four data modality selections, three electrode selections, and seven frequency-band selections, resulting in 44 (2+2 · 3 · 7) result samples per regressor described in sections 4.5, 4.6.

### 4.4. Statistics

In our visualizations and statistics, we focused on aspects which generalize over the whole sample, analysing the regression results pooled over all but the investigated aspect. We use this approach to compensate for the small user sample inherent to a pilot study. Trying to generate results for specific feature, user, and regressor combinations is possible but would most likely not generalize well to a larger user cohort. To evaluate whether a result sample of a given aspect (feature, user, or regressor) significantly differed from its peers, we performed non-parametric tests as our samples were non normal distributed. When pairs were available, we performed two-sided sign-tests using our own implementation. When no pairs were available, e.g., when comparing data selections containing EEG data to data selections without EEG data, we performed two-sided Mann–Whitney-*U*-tests with continuity correction, as implemented in scipy (Jones et al., 2001) version 1.3.0. In both tests, ties correction was performed.

To asses whether the reported regression performances were above chance level, we permuted the subjective ratings of the training set 10^6^ times and compared them to the subjective ratings of the test set using the metrics listed in section 4.2. The length of permuted training subjective ratings was truncated to the length of test subjective ratings after permutation to ensure that our samples were drawn from the entire training subjective ratings distribution. The *p*-values of each regression performance was calculated as

(2) $$\begin{array}{r} p=\frac{n_{permutation\geq regression}+1}{n_{permutations}+1} \end{array}$$

with $n_{permutations}=10^{6}$ and *n*_*permutation*≥*regression*_ the number of permutations having an equal or better performance than the tested regression result.

All calculated *p*-values were corrected for multiple testing using the false discovery rate (FDR) correction for dependent *p*-values (Benjamini and Yekutieli, 2001) as implemented in the multipletests function of statsmodels (Seabold and Perktold, 2010) version 0.9.0. We report the FDR-corrected *p*-values as *q*-values.

### 4.5. CNN Regression

We adapted three CNN classification architectures for regression analysis by removing the softmax layer and applying the mean square error (MSE) loss function to the training. The specific architectures used were (i) a 6-layered CNN (Deep4Net, 4 convolution-pooling blocks), (ii) a 29-layered residual neural network (EEGResNet-29, 13 residual blocks), both described by Schirrmeister et al. (2017), and (iii) a compact CNN (EEGNet V4, 4 layers, 2 convolution-pooling blocks) (Lawhern et al., 2018). These CNN networks were chosen because they have previously been shown to perform well for classification of EEG data. EEGNet and Deep4Net were used as implemented in the Braindecode toolbox (https://github.com/TNTLFreiburg/braindecode/).

The weights of the models were initialized using a uniform distribution as described in Glorot and Bengio (2010). The models were then trained for 200 epochs, using a batch size of 64, a learning rate of 0.001 and a weight decay of 0. As feasibility rather than performance is the main focus of this paper, we use identical hyper-parameters for all models. That the models do learn using these hyper-parameters was verified using the validation set. The input time length of the models was individually adapted so that the predictor time length (data used to compute the MSE loss) was exactly 1 s for all models, irrespective of receptive field size. We use AdamW (Loshchilov and Hutter, 2017) with default parameters as optimizer and schedule the learning rate using cosine annealing (Loshchilov and Hutter, 2016) without restarts. We do not perform early stopping. Instead we use the regressor of the end of the training to predict the ratings, irrespective whether a regressor with better validation accuracy existed during the training.

### 4.6. Non-CNN Regression

We use four regressors implemented in scikit-learn (Pedregosa et al., 2011). A linear regressor, a linear support vector regressor (L-SVR), a non-linear (radial basis function kernel) SVR (RBF-SVR) and a Random Forest regressor (RFR). Using the validation set we adjusted the maximal iterations of the RBF-SVR from infinity to 100,000 and the number of trees of the RFR from 10 to 100. Higher values lead to better validation results for both hyper-parameters but had to be limited because of computational budget. All other parameters were kept to the defaults of scikit-learn version 0.20.2.

### 4.7. Within-User Regression

First, we evaluated the regression on different data modalities within each user. As input data, we either used the position of the robotic hand in 3D, the EEG data, the peripheral physiological data (ECG, respiration), or all combined. Table 3 lists the results of all regressors. As a chance level regression baseline we report the best out of 10^6^ random permutations of the training labels of each user compared to the test labels in Table 3a.

**Table 3:**
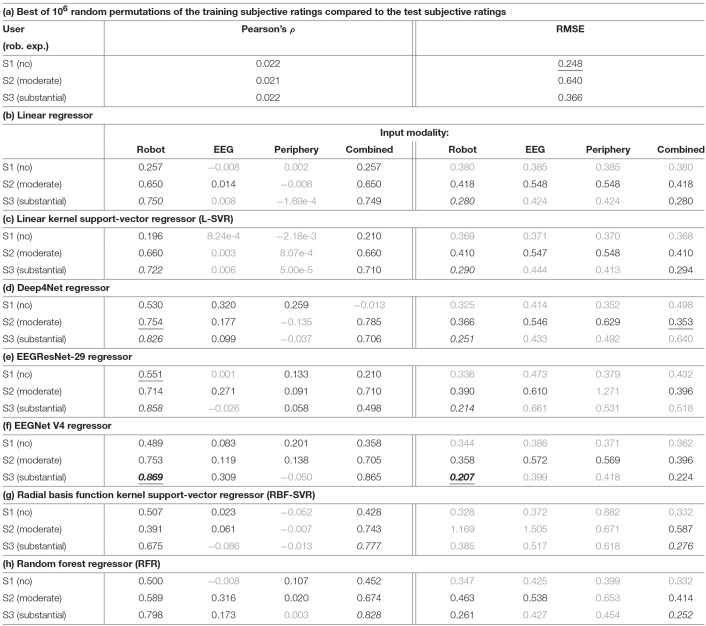
Regression test-set metrics for different data modalities.

*EEG refers to EEG data without any feature selection. Italic entries highlight best results for each regressor. Underlined entries highlight best results for each user. Bold entries highlight overall best results. Gray entries had q ≥ 0.05 when corrected for 28 tests (4 modalities · 7 regressors)*.

Chance levels were not competitive (~0.02) and very similar (±0.001) across users for Pearson's ρ, indicating that the correct shape of the subjective rating cannot be easily guessed. At the same time, the RMSE chance levels were unexpectedly competitive for S1 and S3, confirming that these 2 users rated with lower variance than S2 (0.112 and 0.194 vs. 0.354). No RMSE regression result based on the data of S1 was significantly better than random permutations (q ≥ 0.05).

**Figure 7:**
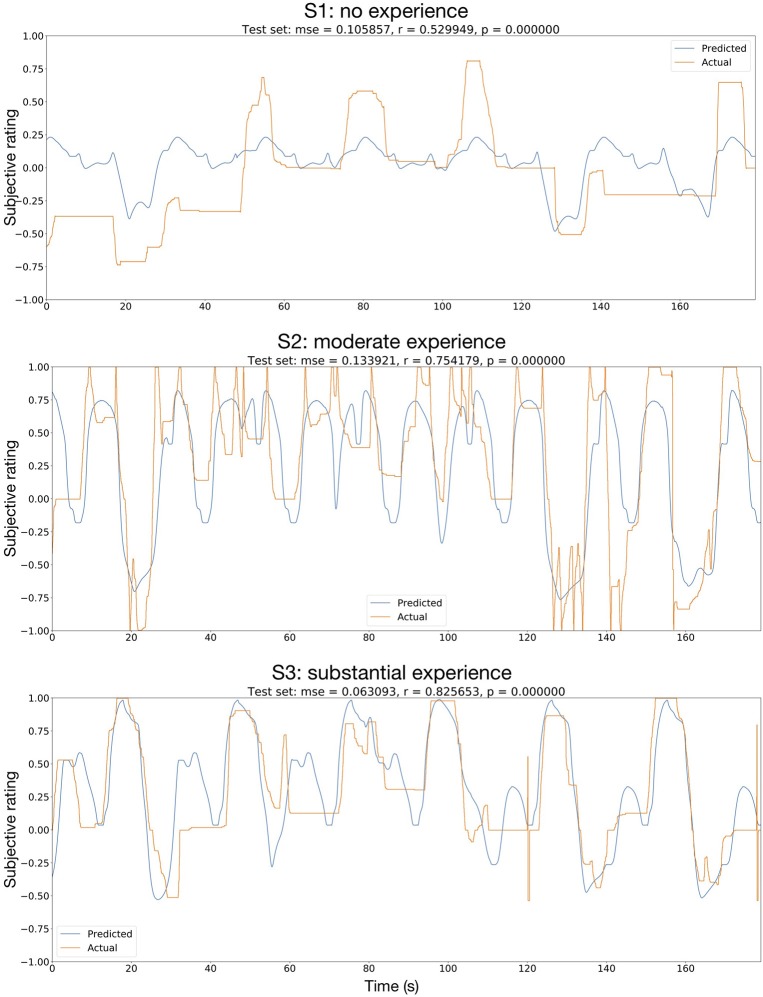
Regression of subjective rating, using the robotic hand position as input. The network used was the Deep4Net, with a predictor time length of 1 s. The orange curve shows the actual rating given by the users, the blue curve shows the predicted rating. mse, mean square error; r, Pearson's ρ; p, *p*-value (uncorrected for multiple testing).

In most cases, the performance in the final evaluation set was the highest with the robotic hand position as the only input data. Predictions from EEG only showed rather low performance, while predictions from ECG & respiration yielded low performance. A combination of all input data did improve the performance in some cases, mostly so for non-linear methods. Predicted and actual rating in the 3-min test set are displayed in Figure 7 for the Deep4Net, trained on the robotic hand position data.

When considering the best results obtained for EEG, periphery data and the combination of all data types, it seems like EEG and periphery data each carry information related to the rating. In the case of the non-linear regressors, this information could be used to improve the regression performance. To investigate the origin of this information, we trained our regressors on different EEG electrode selections and EEG frequency bands.

### 4.8. Regression Using Different EEG Features

**Figure 8:**
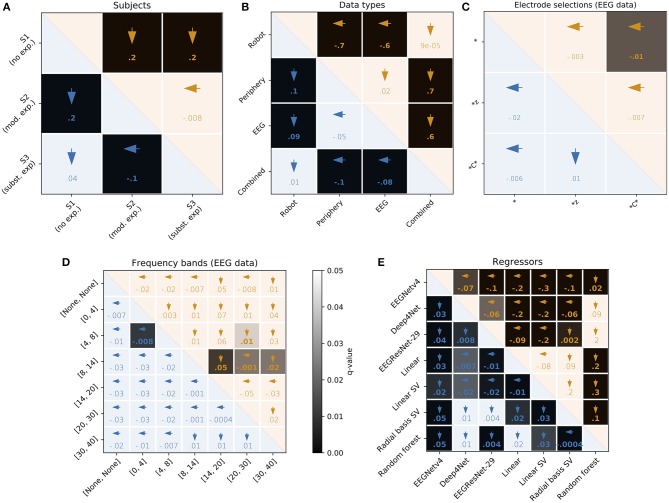
User, feature, and regressor overview. Each matrix is based on the results of the 924 test-set regressions (3 users · 44 features · 7 models), split according to visualized parameter. Numbers in the lower and upper triangles of each comparison matrix are the differences between median RMSE (blue) and Pearson's ρ (orange), respectively. Bold font indicates *q* < 0.05. Arrows indicate the parameter with better performance of each pair. The gray-scale background of each cell codes for the corresponding *q*-value. Separate FDR corrections for each triangle of each matrix. **(A)** Users: FDR-correction for 3 tests. mod., moderate; subst., substantial; exp., experience. **(B)** Data types: FDR-correction for six tests. **(C)** Electrodes. FDR-correction for 3 tests. *All EEG electrodes, *z: midline electrodes, *C*: sensorimotor cortex electrodes. **(D)** Frequency bands: FDR-correction for 21 tests. The corner frequencies of band-pass filters used are indicated in the square brackets. **(E)** Regressor: FDR-correction for 21 tests. SV, support vector.

We trained our regressors on different EEG electrode selections and EEG frequency bands. The differentiation of electrodes and frequency bands has 3 purposes. (1) To investigate the origin of the subjective-rating related information contained in the EEG. (2) To test whether indications from the literature transfer to our paradigm. (3) To probe for a potential motor bias. The results of these regressions are visualized in Figures 8C,D for electrode and frequency-band selections, respectively.

For the electrode selection, the only statistically significant result is that, when considering the shape of the subjective rating (reflected in Pearson's ρ), using all electrodes is marginally better than using only the electrodes located above the sensorimotor cortex (*C*) (*q* = 0.016). Non-significant results when considering shape of the regression are that using all electrodes is marginally better than using only the midline (*z) electrodes (*q* = 0.252) and that using the *C* electrodes is marginally worse than using the *z electrodes (*q* = 0.130). When considering the values of the regressions (reflected by the RMSE), non-significant results are that using all electrodes is marginally worse than using both *z electrodes (*q* = 1) and *C* electrodes (*q* = 1) and that using *z electrodes is marginally better than using *C* electrodes (*q* = 1). Summarizing, no considerable effect could be found. It appears like using all electrodes is marginally best when considering the shape of the regression and using *z electrodes is marginally best when considering the values of the regression.

For the frequency-band selections, four comparisons related to the shape of the regression and one comparison related to the values of the regressions were statistically significant. Using the low-beta (14–20 Hz) and high-beta/low-gamma (30–40 Hz) frequency bands gave marginally better (higher) correlation coefficients than using the alpha (8–14 Hz) band (*q* = 0.009 and 0.023, respectively). Using the alpha band was marginally better than using the mid-beta (20–30 Hz) band (*q* = 0.023). Using the mid-beta band was marginally better than using the theta (4–8 Hz) band (*q* = 0.043). Finally, the RMSE was marginally better (lower) using the theta band than using the delta (0–4 Hz) band. The general tendency seen in the statistically significant results, higher frequency bands providing better results than lower frequency bands, can also be seen in the non-significant results. Similar to the electrode selection, no considerable effect was found.

**Figure 9:**
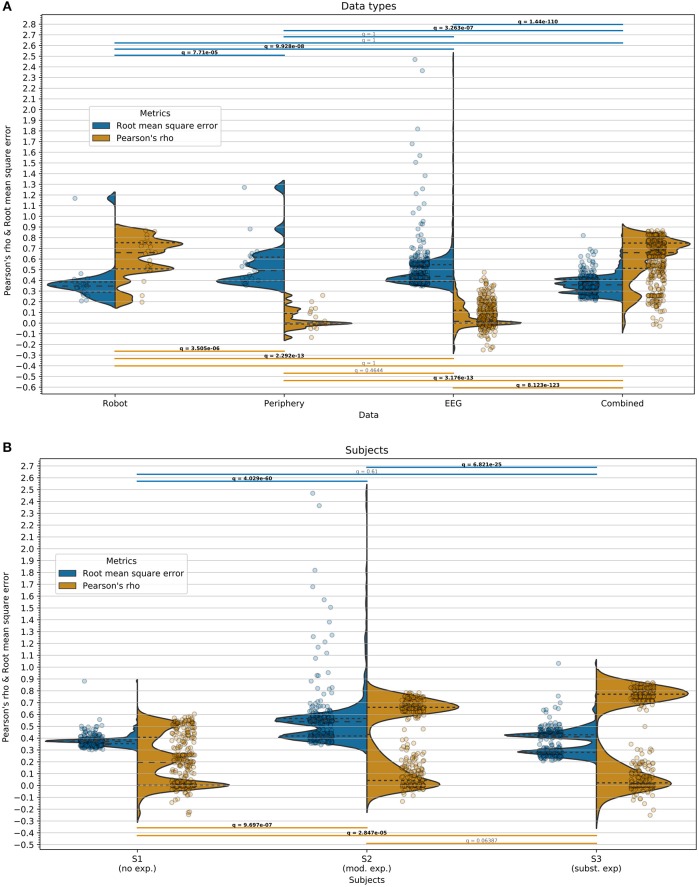
Regression within users. Pearson's ρ (orange, higher is better) and the RMSE (blue, lower is better) are displayed separately in split violins. As test-set, the last 3 min of the experiment was used for each user. The dashed lines within the split violins represent the quartiles (from bottom to top, 25th, 50th, and 75th percentiles, respectively). Overlaid to the split violins are dot-plots with horizontal jitter (for better visibility) representing the data sample underlying the split violins. The horizontal bars above and below the split violins indicate the performed significance tests with matching q-value (FDR-corrected *p*-value). Bold font indicates *q* < 0.05. The evaluation metrics with their corresponding half-violins, dot-plots, and significance bars are color-coded. **(A)** Regression results split across data types: 21 or 441 test-set regressions per split violin (3 users · 1 features · 7 models or 3 users · 21 feature · 7 models). Significance (unpaired: Mann–Whitney-U, paired: sign-test) FDR-corrected for 6 tests. Separate corrections for each metric. **(B)** Regression results split across users: 308 test-set regressions (44 features · 7 models) per split violin. Significance (sign-test) FDR-corrected for 3 tests. Separate corrections for each metric.

An overview of the regression results across all within-user regressions can be found in Figures 8A, 9B to compare users, Figures 8B, 9A to compare data types and Figure 8E to compare regressors. An overview of the across-user regression results is provided in the next section.

### 4.9. Across-User Regression

**Figure 10:**
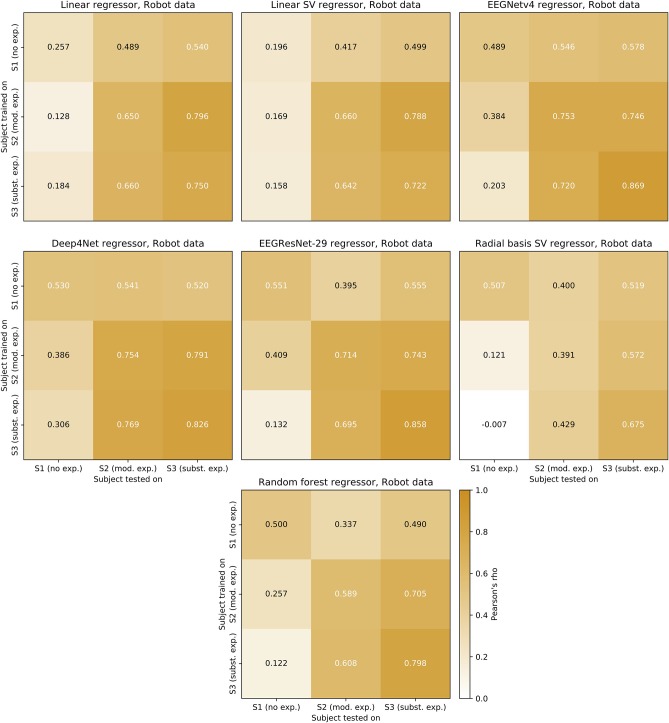
Across-user transfer matrices. Regression across users trained on the position of the robotic hand. As test-set, the last 3 min of the experiment was used for each user. The transfer matrix displays the user on which the regressor was trained (y-axis) and the user on which it was evaluated (x-axis). The evaluation metric (Pearson's ρ) is displayed color-coded and in text within the respective field.

We further applied the regressors trained on the robot data across users, to test for user-specific differences the regressors might have learned. The results are shown in Figure 10. We now only consider Pearson's ρ as our performance metric.

A pattern common to all regressors was that models trained on a user with less experience performed similarly or better when tested to a user with more experience, relative to within-user. For example, training on S2 and testing on S3 resulted in higher correlations in 6 out of 7 regressors, relative to testing on S2. The 7th regressor (EEGNet v4) performed similarly (0.007 difference) when trained on S2 and tested on both S2 and S3. Training on a user with more experience and testing on a user with less experience, reversed the pattern. For example, training on S3 and testing on S1 and S2 always resulted in lower correlations than when testing on S3. Increases and decreases in performance scaled with the experience in 36 out of 42 transfers.

Interestingly, performance gains relative to within-user regression could be achieved when transferring between S2 and S3 for some regressors. This was not the case for all regressors when transferring from S2 and S3 to S1. That is, training on S2 and testing on S3 resulted in better performance than within-user regression in S3 for both linear regressors. Similarly, training on S3 and testing on S2 resulted in better performance than within-user regression in S2 in 4 out of 7 regressors. Furthermore, the best performance in S2 was achieved by transferring from S3 using a CNN regressor (Deep4Net). For S1 and S3, best performance was achieved within-user (EEGRestNet-29 and EEGNet v4, respectively).

## 5. Discussion

The main contribution of the present study is to describe and evaluate a novel method for continuous rating of subjective user perception during direct human-robot interaction. Our rating approach is continuous in two respects: first, ratings are conveyed on a continuous scale, secondly, these ratings are acquired continuously over the whole time period during which users interact with the robotic system. This rating system was designed to minimize movement-related confounding effects (see section 5.4), and as we have demonstrated, allows to generate real-time user feedback during a grasp-and-deliver task of a robotic arm by means of a wireless controller. We have used our rating system to let users evaluate the general behavioral quality of the robot the users were interacting with, ranging from “positive” to “negative.” However, the rating system is amendable to any other continuous variable, and would thus also allow rating of factors like subjective valence and arousal, or trustworthiness of a robot, as in Sarkar et al. (2017).

### 5.1. Subjective Ratings

In the present study, we chose a scenario inspired by emerging brain-computer interfacing (BCI) applications. While our main focus was on demonstrating the basic feasibility and usefulness of our continuous rating system, the data that we obtained during the evaluation process in three users with different levels of previous direct experience with robotic systems lead to several preliminary observations that provide potentially useful starting points for further large scale investigations. As described in 3 in more detail, the three users showed distinct rating patterns with, for example, temporally more stable rating behavior in the users with more extensive previous robot exposure (Figure 6). Of course, based on the current data, we cannot decide whether it was experience *per se* or possibly pre-existing personality traits such as anxiety, or a more or less positive basic attitude and trust in technology in general, and robots in particular, that had a modulating influence on the inter-individual differences that we have observed. This line of interpretation would also fit to the results demonstrated by Sarkar et al. (2017). There, users with more experience rated the robots as less intelligent and less safe than their counterparts with less experience. Such and related questions could be addressed using experimental procedures with fine-grained rating systems as we have described here.

### 5.2. Regression of Subjective Ratings

While explicit user ratings can be useful, in many situations it would be inconvenient or even impossible to obtain such ratings. For example, in a BCI scenario, as investigated here, where paralyzed patients may entirely loose the ability to convey motor responses. Implicit (based on physiological measurements from the users) or objective/contextual sources of information (based on the robot behavior or environmental factors) could help in such situations. We thus tested whether various neuro- and peripheral recordings (EEG, ECG, respiration) as well as kinematic properties of the robot actions contained information about the subjective ratings. As we did not observe strong linear correlations, we applied diverse regression methods to predict subjective ratings. At least based on the data available here, robot hand position was the best predictor for the subjective ratings, reaching correlation coefficients up to 0.869.

Robot hand position based performance of the different regressors revealed a consistent ranking of the users. According to correlation coefficient, user rank scaled with experience. This could be interpreted as a sign that more experienced users produce subjective ratings which are more consistent over time and thus provide a better generalization form training to testing. The aspect of rating consistency was also discussed in 5.1. There, we noted that due to the small sample size of our pilot cohort other personal traits influencing rating consistency can not be ruled out. Additionally, the amount of training data also scaled with experience (cf. section 2.2), which could have biased the regression performance. Finally, according to rmse, user rank no longer scaled with experience, further supporting our caution.

Dry EEG and peripheral physiological recordings were not very useful for the regression analysis. Only weak correlation values between predicted and actual rating up to 0.320 could be achieved in the final evaluation sets. A combination of different data modalities also did not improve the prediction beyond that obtained with the robot position as the only input. Dry EEG as used here has a generally lower signal-to-noise ratio than conventional EEG (Mathewson et al., 2017), and the experiments were conducted in a high-noise setting. Thus, in future, improved dry EEG recording techniques (Fiedler et al., 2015) or gel-filled EEG electrodes applied in recording conditions optimized for high EEG signal quality (Völker et al., 2018a) could help to evaluate the full potential of EEG in the present context. Further peripheral physiological signals such as electrodermal activity (EDA), as an index of arousal and stress (Fowles, 1980), could also be tested in future studies.

### 5.3. EEG Signals Related to Subjective Ratings

In a preliminary attempt to identify EEG signals related to the subjective ratings, we investigated the selection of EEG electrodes and frequency bands. As reported in section 4.8, it appeared like using all electrodes was marginally but partially statistically significantly best when considering the shape of the regression and using *z electrodes was marginally but non-significantly best when considering the values of the regression (cf. Figure 8C). The latter would be in line with reports form the literature highlighting the importance of *z electrodes for detecting error-related brain activity (Cavanagh et al., 2010; Cavanagh and Frank, 2014; Spüler and Niethammer, 2015; Völker et al., 2018a). Unfortunately, we could not find further evidence relating our results to the literature as we could not show that the delta and theta bands play a dominant role in the encoding of error-related brain activity in our paradigm. Rather, frequencies in the alpha and beta bands seemed to be more informative, albeit only partially significantly (cf. Figure 8D). At the moment it is still unclear whether this trend is related to error signals or a motor bias.

### 5.4. Motor Bias

In order to be compatible with EEG, we designed the experimental paradigm in such a way that each evaluation value corresponds to an equal tonic motor output. However, directional motor signals (Waldert et al., 2009) could potentially still confound the interpretation of EEG signals correlated with subjective ratings. No evidence of a motor bias was found using the electrode selection as the *C* performed consistently worst. However, one would have expected to observe improved results when using the alpha and beta bands relative to the delta and theta bands, with a dominance of the alpha band. Indeed, alpha and beta bands were the best bands when considering non-significant RMSE results but with a dominance of the mid-beta band. In the partially significant Pearson's ρ results, the low-beta band dominated, which is not entirely typical of a motor bias, especially relative to the alpha band (Pfurtscheller and Lopes da Silva, 1999). At this point, a motor bias cannot be entirely ruled out based on the frequency-band selection. More experiments and data analysis, for example by contrasting the EEG data during extremely negative with extremely positive subjective ratings, will be needed to resolve this question. Future studies should explicitly control for motor bias, e.g., by switching the vertical or horizontal axis of the rating system, either between measurement sessions or between users.

### 5.5. Across-User Regression of Subjective Ratings

To investigate whether the models have learned user-specific characteristics, we have applied the models trained on the training set of one user to the test sets of all users. In section 4.9 we have described the general patterns underlying our initial across-user regression results. Based on our 3 pilot users, we found that the user's prior experience with robots might also play a role when transferring models between users. Models trained on users with less experience mostly increased their performance when transferred to users with more experience (in 15 out of 21 transfers) and *vice versa* (in 21 out of 21 transfers). The more experience the transferred to user had the better the correlation coefficient and *vice versa* (in 36 out of 42 transfers). As the best across-user transfers always performed better on the tested user but only rarely surpassed the within-user test on the training user, our findings hint at the possibility that regressors have learned both user-specific and general characteristics. This seems to make it possible to generalize across users in decoding of subjective perception from kinematic data of the robot actions. At the same time, the results indicate that a good set of training data from which the CNN is able to generalize well might be more important than the user-specific differences of the users.

In addition to our small sample size and differences in the amount of training data, it is here also important to consider that, as discussed in section 5.2, within-user performance (Pearson's ρ) appeared to scale with experience, which might be a confounding factor here. If the reported patterns still hold for larger samples, disentangling the factors, if possible, will be necessary to differentiate their contributions to the reported across user transfer effect. It will also be interesting to investigate whether the across-user performance can be improved beyond the within-user performance by combining the data of additional users in the across-user training. Including the training data of the tested user into a hybrid within/across-user training could also further improve regression performance. Although such data would not be directly available in an online BCI scenario an initial across-user model could be fine-tuned as more and more labeled data of a new user become available during an online experiment as e.g., we have done for online-adaptive classification using CNNs in Kuhner et al. (2019).

## 6. Outlook

The connection of subjective continuous ratings in real human-robot interaction with EEG allows the search for neurophysiological correlates of these ratings, which could then be used as features for automated behavior adaptation algorithms during robot interaction with paralyzed patients. Even if such a search would fail, the availability of continuous ratings would make it possible to generate fine-grained robotic behavior policies, which in turn could be used to improve robot behavior. In the future, the availability of continuous rating data may be useful both in *post-hoc* and real-time application scenarios. For example, *post-hoc* analysis of continuous robot-related user rating would allow to study how different aspects of robot behavior may shape user perception, and how user perception evolves over time. Real-time analysis of ratings could convey important teaching signals for real-time adaptation and personalization of robot behavior, for example for users with different levels of previous exposure to robots. Thus in the future, real-time ratings combined with reinforcement-learning methods, e.g., Deep Q-Networks (DQNs, Mnih et al., 2015) or Deep Deterministic Policy Gradient algorithms (DDPG, Lillicrap et al., 2015), could enable robot systems that keep optimizing their behavior in a human-compliant manner.

## Ethics Statement

This study was carried out in with written informed consent from all subjects. All subjects gave written informed consent in accordance with the Declaration of Helsinki. The protocol was approved by the Ethics Committee, University of Freiburg.

## Author Contributions

TB, JB, WB, MV, and LF contributed conception and design of the study. RS provided code and advice. MV and LF wrote code for the experiments and analyses. MV performed the EEG recordings and simulations. TB, MV, and LF wrote sections of the manuscript.

### Conflict of Interest

The authors declare that the research was conducted in the absence of any commercial or financial relationships that could be construed as a potential conflict of interest.

## References

[B1] Bashivan P. Rish I. Yeasin M. Codella N. (2016). Learning representations from EEG with deep recurrent-convolutional neural networks. ArXiv e-prints.

[B2] Benjamini Y. Yekutieli D. (2001). The control of the false discovery rate in multiple testing under dependency. Ann. Stat. 29, 1165–1188. 10.1214/aos/1013699998

[B3] Burget F. Fiederer L. D. J. Kuhner D. Völker M. Aldinger J. Schirrmeister R. T. (2017). Acting thoughts: towards a mobile robotic service assistant for users with limited communication skills, in 2017 European Conference on Mobile Robots (ECMR) (Paris), 1–6.

[B4] Cavanagh J. F. Frank M. J. (2014). Frontal theta as a mechanism for cognitive control. Trends Cogn. Sci. 18, 414–421. 10.1016/j.tics.2014.04.012 24835663 PMC4112145

[B5] Cavanagh J. F. Frank M. J. Klein T. J. Allen J. J. B. (2010). Frontal theta links prediction errors to behavioral adaptation in reinforcement learning. NeuroImage 49, 3198–3209. 10.1016/j.neuroimage.2009.11.080 19969093 PMC2818688

[B6] Chitta S. Sucan I. Cousins S. (2012). Moveit![ros topics]. IEEE Robot. Autom. Mag. 19, 18–19. 10.1109/MRA.2011.2181749

[B7] Ehrlich S. K. Cheng G. (2018). A feasibility study for validating robot actions using EEG-based error-related potentials. Int. J. Soc. Robot. 11, 1–13. 10.1007/s12369-018-0501-8

[B8] Eitel A. Springenberg J. T. Spinello L. Riedmiller M. Burgard W. (2015). Multimodal deep learning for robust rgb-d object recognition, in 2015 IEEE/RSJ International Conference on Intelligent Robots and Systems (IROS) (Hamburg: IEEE), 681–687.

[B9] Feil-Seifer D. Skinner K. Matarić M. J. (2007). Benchmarks for evaluating socially assistive robotics. Interact. Stud. 8, 423–439. 10.1075/is.8.3.07fei

[B10] Fiedler P. Griebel S. Pedrosa P. Fonseca C. Vaz F. Zentner L. (2015). Multichannel eeg with novel ti/tin dry electrodes. Sensors Actuat. A Phys. 221, 139–147. 10.1016/j.sna.2014.10.010

[B11] Fowles D. C. (1980). The three arousal model: implications of gray's two-factor learning theory for heart rate, electrodermal activity, and psychopathy. Psychophysiology 17, 87–104. 10.1111/j.1469-8986.1980.tb00117.x 6103567

[B12] Glorot X. Bengio Y. (2010). Understanding the difficulty of training deep feedforward neural networks, in Proceedings of the Thirteenth International Conference on Artificial Intelligence and Statistics, Vol. 9 of *Proceedings of Machine Learning Research*, eds Teh Y. W. Titterington M. (Sardinia), 249–256.

[B13] Held D. Thrun S. Savarese S. (2016). Learning to track at 100 fps with deep regression networks, in European Conference on Computer Vision (Amsterdam: Springer), 749–765.

[B14] Huang C.-M. Mutlu B. (2012). Robot behavior toolkit: generating effective social behaviors for robots, in 2012 7th ACM/IEEE International Conference on Human-Robot Interaction (HRI) (Boston, MA: IEEE), 25–32.

[B15] Iturrate I. Montesano L. Minguez J. (2013). Shared-control brain-computer interface for a two dimensional reaching task using EEG error-related potentials, in 2013 35th Annual International Conference of the IEEE Engineering in Medicine and Biology Society (EMBC) (Osaka: IEEE), 5258–5262. 10.1109/EMBC.2013.6610735 24110922

[B16] Jones E. Oliphant T. Peterson P. (2001). SciPy: Open Source scientific Tools for Python. Vienna 10.1145/3029798.3034826

[B17] Kolkhorst H. Tangermann M. Burgard W. (2017). Decoding perceived hazardousness from user's brain states to shape human-robot interaction, in Proceedings of the Companion of the 2017 ACM/IEEE International Conference on Human-Robot Interaction (Vienna: ACM), 349–350.

[B18] Kolkhorst H. Tangermann M. Burgard W. (2018). Guess what i attend: interface-free object selection using brain signals, in 2018 IEEE/RSJ International Conference on Intelligent Robots and Systems (IROS) (Madrid: IEEE).

[B19] Kuhner D. Fiederer L. D. J. Aldinger J. Burget F. Völker M. Schirrmeister R. T. (2019). A service assistant combining autonomous robotics, flexible goal formulation, and deep-learning-based brain-computer interfacing. Robot. Auton. Syst. 116, 98–113. 10.1016/j.robot.2019.02.015

[B20] Lawhern V. Solon A. Waytowich N. Gordon S. M. Hung C. Lance B. J. (2018). EEGnet: a compact convolutional neural network for EEG-based brain–computer interfaces. J. Neural Eng. 15:056013. 10.1088/1741-2552/aace8c 29932424

[B21] Lillicrap T. P. Hunt J. J. Pritzel A. Heess N. Erez T. Tassa Y. (2015). Continuous control with deep reinforcement learning. arXiv [Preprint]. arXiv:1509.02971.

[B22] Loshchilov I. Hutter F. (2016). SG DR: stochastic gradient descent with warm restarts. arXiv:1608.03983.

[B23] Loshchilov I. Hutter F. (2017). Fixing weight decay regularization in Adam. arXiv:1711.05101.

[B24] Manor R. Geva A. B. (2015). Convolutional neural network for multi-category rapid serial visual presentation BCI. Front. Comput. Neurosci. 9:146. 10.3389/fncom.2015.00146 26696875 PMC4667102

[B25] Mathewson K. E. Harrison T. J. Kizuk S. A. (2017). High and dry? Comparing active dry eeg electrodes to active and passive wet electrodes. Psychophysiology 54, 74–82. 10.1111/psyp.12536 28000254

[B26] Miao S. Wang Z. J. Liao R. (2016). A cnn regression approach for real-time 2D/3D registration. IEEE Trans. Med. Imaging 35, 1352–1363. 10.1109/TMI.2016.2521800 26829785

[B27] Mnih V. Kavukcuoglu K. Silver D. Rusu A. A. Veness J. Bellemare M. G.. (2015). Human-level control through deep reinforcement learning. Nature 518:529. 10.1038/nature14236 25719670

[B28] Oliveira G. L. Valada A. Bollen C. Burgard W. Brox T. (2016). Deep learning for human part discovery in images, in 2016 IEEE International Conference on Robotics and Automation (ICRA) (Stockholm: IEEE), 1634–1641.

[B29] Paszke A. Gross S. Chintala S. Chanan G. Yang E. DeVito Z. (2017). Automatic differentiation in PyTorch, in NIPS Autodiff Workshop. Long Beach.

[B30] Pedregosa F. Varoquaux G. Gramfort A. Michel V. Thirion B. Grisel O. (2011). Scikit-learn: machine learning in Python. J. Mach. Learn. Res. 12, 2825–2830.

[B31] Pfurtscheller G. Allison B. Z. Bauernfeind G. Brunner C. Solis Escalante T. Scherer R.. (2010). The hybrid BCI. Front. Neurosci. 4:3. 10.3389/fnpro.2010.00003 20582271 PMC2891647

[B32] Pfurtscheller G. Lopes da Silva F. H. (1999). Event-related EEG/MEG synchronization and desynchronization: basic principles. Clin. Neurophysiol. 110, 1842–1857. 10.1016/S1388-2457(99)00141-8 10576479

[B33] Quigley M. Conley K. Gerkey B. Faust J. Foote T. Leibs J. (2009). Ros: an open-source robot operating system, in ICRA Workshop on Open Source Software, Vol. 3 (Kobe).

[B34] Rohmer E. Singh S. P. Freese M. (2013). V-rep: A versatile and scalable robot simulation framework, in 2013 IEEE/RSJ International Conference on Intelligent Robots and Systems (IROS) (Tokyo: IEEE), 1321–1326.

[B35] Salazar-Gomez A. F. DelPreto J. Gil S. Guenther F. H. Rus D. (2017). Correcting robot mistakes in real time using EEG signals, in 2017 IEEE International Conference on Robotics and Automation (ICRA) (Singapore), 6570–6577.

[B36] Sarikaya D. Corso J. J. Guru K. A. (2017). Detection and localization of robotic tools in robot-assisted surgery videos using deep neural networks for region proposal and detection. IEEE Trans. Med. Imaging 36, 1542–1549. 10.1109/TMI.2017.2665671 28186883

[B37] Sarkar S. Araiza-Illan D. Eder K. (2017). Effects of faults, experience, and personality on trust in a robot co-worker. arXiv [Preprint]. arXiv:1703.02335.

[B38] Schalk G. McFarl D. J. Hinterberger T. Birbaumer N. Wolpaw J. R. (2004). BCI2000: a general-purpose brain-computer interface (BCI) system. IEEE Trans. Biomed. Eng. 51:2004. 10.1109/TBME.2004.827072 15188875

[B39] Schirrmeister R. T. Springenberg J. T. Fiederer L. D. J. Glasstetter M. Eggensperger K. Tangermann M.. (2017). Deep learning with convolutional neural networks for EEG decoding and visualization. Hum. Brain Mapp. 38, 5391–5420 10.1002/hbm.23730 28782865 PMC5655781

[B40] Seabold S. Perktold J. (2010). Statsmodels: econometric and statistical modeling with python, in 9th Python in Science Conference. Austin.

[B41] Sekmen A. Challa P. (2013). Assessment of adaptive human–robot interactions. Knowl. Based Syst. 42, 49–59. Seabold and Perktold, 2010

[B42] Shi B. Bai X. Liu W. Wang J. (2016). Face alignment with deep regression. IEEE Trans. Neural Netw. Learn. Syst. 28, 183–194. 10.1109/TNNLS.2016.2618340 27831893

[B43] Spüler M. Niethammer C. (2015). Error-related potentials during continuous feedback: using EEG to detect errors of different type and severity. Front. Hum. Neurosci. 9:155. 10.3389/fnhum.2015.00155 25859204 PMC4374466

[B44] Tapus A. Ţăpuş C. Matarić M. J. (2008). User? Robot personality matching and assistive robot behavior adaptation for post-stroke rehabilitation therapy. Intell. Service Robot. 1:169 10.1007/s11370-008-0017-4

[B45] Viereck U. Pas A. t. Saenko K. Platt R. (2017). Learning a visuomotor controller for real world robotic grasping using simulated depth images. arXiv preprint arXiv:1706.04652.

[B46] Völker M. Fiederer L. D. J. Berberich S. Hammer J. Behncke J. Kršek P.. (2018a). The dynamics of error processing in the human brain as reflected by high-gamma activity in noninvasive and intracranial EEG. NeuroImage 173, 564–579. 10.1016/j.neuroimage.2018.01.059 29471099

[B47] Völker M. Hammer J. Schirrmeister R. T. Behncke J. Fiederer L. D. J. Schulze-Bonhage A. (2018b). Intracranial error detection via deep learning, in IEEE International Conference on Systems, Man, and Cybernetics (SMC) (Miyazaki).

[B48] Völker M. Schirrmeister R. T. Fiederer L. D. J. Burgard W. Ball T. (2018c). Deep transfer learning for error decoding from non-invasive EEG, in IEEE 6th International Conference on Brain-Computer Interface (BCI) (High1-gil).

[B49] Waldert S. Pistohl T. Braun C. Ball T. Aertsen A. Mehring C. (2009). A review on directional information in neural signals for brain-machine interfaces. J. Physiol. 103, 244–254. 10.1016/j.jphysparis.2009.08.007 19665554

[B50] Watter M. Springenberg J. Boedecker J. Riedmiller M. (2015). Embed to control: a locally linear latent dynamics model for control from raw images, in Advances in Neural Information Processing Systems (Montreal), 2746–2754.

